# PReS-FINAL-2178: Clinical and microarray follow-up of SJIA patients treated with anakinra over the past 10 years in a single institution

**DOI:** 10.1186/1546-0096-11-S2-O13

**Published:** 2013-12-05

**Authors:** M Punaro, A Gotte, M Stoll, D Blankenship, F Allantaz, Z Xu, V Pascual

**Affiliations:** 1 Arthritis, Texas Scottish Rite Hospital for Children, Dallas, TX, USA; 2 Arthritis, Baylor Institute of Immunology Research, Dallas, TX, USA; 3 Research, Baylor Institute of Immunology Research, Dallas, TX, USA

## Introduction

The role of IL-1 in the pathogenesis of SJIA was first reported by our group in 2005.

## Objectives

To evaluate the response to IL-1 blockade over the past 10 years in sJIA patients treated with anakinra at the clinical level including the durability of response, long term complications, and steroid sparing effect as well as to utilize blood gene expression profiling for insight into potential mechanisms of pathogenesis.

## Methods

Clinical/laboratory data of all children with sJIA at our institution treated with anakinra with at least 6 months of follow-up were reviewed. Whole blood gene expression profiling (Illumina bead chip array) was obtained in most patients before and after initiation of IL-1 blockade and repeated over the course of follow-up.

## Results

51 SOJIA patients (30 F/21 M) with median disease duration of 1.0 years (range 0 days post dx-11.6 years) and median of 4 active joints (range 0-40) at initiation of anakinra were treated with a mean dose of 2.67 mg/kg (range 1-10) with an average follow-up of almost 5 years (range 0.49-9.75) on anakinra. All children had a sJIA signature as previously described (1) by microarray analysis.

After 1L-1 blockade, significant improvements were seen in rash (p = 0.0008), fever (p < 0.0001), number of active joints (p = 0.0002), WBC (p < 0.0001), hemoglobin (p < 0.0001), platelets (p < 0.00001), and ESR (p < 0.0001).

Twenty-two patients (17 new, 5 with flares) were treated without any steroids on anakinra alone (18), or concomitantly with methotrexate (4).

37/51 (72%) patients met Wallace criteria for inactive disease, 30/51 (59%) for clinical remission on medications, and 13/51 (25%) met criteria for clinical remission. Eight children (16%) had a partial response with important clinical improvements and were able to stop or greatly wean steroids. Five children (10%) had no sustained response.

Most side effects were minor although 1 patient had sepsis immediately after IVMP. One patient had a drug induced hepatitis necessitating discontinuation of anakinra. One patient continued anakinra throughout pregnancy and delivered a normal term baby. Four patients with clinical MAS have resolved on anakinra.

Gene expression profiling showed a remarkably homogeneous pattern of IL-1 related gene dysregulation (1), which normalized in most patients after anakinra. In some patients there was a time lapse between clinical response and correction of gene expression, suggesting that immune alterations are not completely resolved at time of first clinical response. Most upregulated transcripts encoded innate immunity related proteins. Down regulated transcripts encoded proteins involve in cytoxic/NK cell function and protein synthesis. In a small number of patients, interferon inducible genes were upregulated, especially in patient who had never received steroids and 5/7 of these showed normalization post anakinra.

## Conclusion

Anakinra is safe and effective in controlling clinical disease and correcting gene expression alterations in most children with sJIA in long term follow up. When used early in the disease course, most patients can be managed without corticosteroids, historically a major cause of morbidity in these patients.

## Disclosure of interest

None declared.

